# Differential associations of angiographic extent and severity of coronary artery disease with asymmetric dimethylarginine but not insulin resistance in non-diabetic men with stable angina: a cross-sectional study

**DOI:** 10.1186/1475-2840-12-145

**Published:** 2013-10-09

**Authors:** Olga Kruszelnicka, Andrzej Surdacki, Alain Golay

**Affiliations:** 1 Department of Coronary Artery Disease, The John Paul II Hospital, 80 Prądnicka Street, Cracow 31-202, Poland; 2 2nd Department of Cardiology, Jagiellonian University Medical College and University Hospital, 17 Kopernika Street, Cracow 31-501, Poland; 3 Division of Therapeutic Education for Chronic Diseases, Geneva University Hospitals, 24 rue Micheli-du Crest, 1211-Geneva-14, Switzerland

**Keywords:** ADMA, Coronary artery disease, Insulin resistance

## Abstract

**Background:**

Asymmetric dimethylarginine (ADMA), an endogenous nitric oxide synthesis inhibitor, and insulin resistance (IR) have been implicated in atherogenesis. Our aim was to estimate relations between ADMA, the magnitude of IR and angiographic indices of extent and severity of coronary atherosclerosis in non-diabetic men with stable coronary artery disease (CAD).

**Methods:**

We studied 151 non-diabetic men (mean age 57 ± 11 years) with stable angina, obstructive CAD (at least 1 luminal diameter stenosis of ≥70% in major coronary segments) and without heart failure, and 34 age-matched controls free of ≥50% coronary narrowings. The following CAD indices were computed: the number of major epicardial vessels with ≥70% stenosis, Sullivan extent score representing a proportion of the visible coronary tree with vessel wall irregularities, and Gensini score which reflects both CAD severity and extent, yet assigning a heavier weight to proximal segments and to the more severe narrowings by a non-linear point system. An estimate of IR was derived by homeostasis model assessment (HOMA-IR) from fasting insulin and glucose.

**Results:**

Among the CAD patients, the proportions of subjects with 1-vessel, 2- vessel and 3-vessel CAD were 26%, 25% and 49%, respectively. ADMA levels were higher in patients with obstructive CAD compared to the controls (0.51 ± 0.10 vs. 0.46 ± 0.09 μmol/L [SD], *P* = 0.01), whereas HOMA-IR was similar (median, 3.2 [interquartile range: 2.4–4.9] vs. 2.9 [2.3–4.7], *P* = 0.2). Within the CAD group, ADMA increased across ascending quartiles of Sullivan score (Spearman’s rho = 0.23, *P* = 0.004), but not with Gensini score (rho = 0.12, *P* = 0.15) or the number of vessels involved (rho = 0.08, *P* = 0.3). ADMA correlated to log-transformed Sullivan score (Pearson's *r* = 0.21, *P* = 0.008), which was only slightly attenuated upon multivariate adjustment (*β* = 0.19 ± 0.08 [SEM], *P* = 0.015). HOMA-IR did not differ according to any measure of angiographic CAD (*P* ≥ 0.2). ADMA and log (HOMA-IR) were mutually unrelated (*r* = 0.07, *P* = 0.4).

**Conclusions:**

ADMA is associated with diffuse but not focal coronary atherosclerosis in non-diabetic men with stable CAD irrespectively of the degree of IR. The independent relationship between ADMA and coronary atherosclerotic burden may contribute to the well-recognized prognostic effect of ADMA in CAD.

## Background

Asymmetric dimethylarginine (ADMA) – an endogenous inhibitor of nitric oxide (NO) formation – has been implicated in atherogenesis [1,2]. Nevertheless, despite a bulk of evidence linking ADMA to subclinical carotid atherosclerosis [3-5] and adverse cardiovascular (CV) events [6-10], there are inconsistent reports on the association between ADMA and angiographic coronary artery disease (CAD). Admittedly, the seminal publication by Lu et al. [11] and some other studies [10,12,13] suggested independent relations between elevated ADMA and the presence or extent of angiographic CAD. However, according to the largest reports on ADMA levels in predominantly stable CAD, positive correlations between ADMA and angiographic CAD either were absent [14] or lost significance on multivariate adjustment [8,9], in contrast to detrimental prognostic effects of ADMA on CV outcome [8,9,14].

The magnitude of insulin resistance (IR) has been linked to the incidence of symptomatic CV disease and adverse outcome [15-19]. It is noteworthy that, using a mathematical model, Eddy et al. [20] recently estimated that IR was the most important single cause of symptomatic CAD being responsible for about 42% of myocardial infarctions. Nevertheless, like with ADMA, reports on the relationship of angiographic CAD with IR or its surrogate measure, fasting insulinemia, are discordant with negative findings obtained in larger study groups [21-29]. In addition, there are inconsistent reports on the association between IR and ADMA levels in various clinical settings [30-43].

To the best of our knowledge, exclusively two studies examined the relationship of angiographic CAD with both IR and ADMA. First, our group had previously described independent and additive effects of a hyperinsulinemic euglycemic clamp-derived index of IR and an increased ADMA to L-arginine ratio, but not ADMA, on the number of major coronary vessels with >50% narrowings in 53 patients with stable angina and pre-diabetes [35]. Second, Isiklar et al. [41] have recently observed the lack of correlation between ADMA and Gensini score in 75 stable angina subjects, including 17 type 2 diabetics. Nevertheless, ADMA was unrelated to the degree of IR in the both cited studies [35,41].

Therefore, in order to differentiate between the effects of ADMA and IR on coronary atherosclerosis, we investigated mutual relations between plasma ADMA, the degree of IR and angiographic indices of CAD extent and severity in non-diabetic men with stable CAD.

## Methods

### Subjects

We studied 151 non-diabetic men (mean age 57 ± 11 years) with stable CAD and significant epicardial coronary narrowings (at least 1 luminal diameter stenosis of ≥70%) who underwent an elective coronary angiography. The current report has been based on the extension of the previously described group of 80 CAD patients free of diabetes [43]. In addition, we recruited 34 control men (matched for age and body-mass index) among patients with suspected CAD who exhibited no coronary diameter narrowings ≥50%.

Beyond diabetes (diagnosed according to the 2003 recommendations of the American Diabetes Association by either fasting glucose or postload glycemia during an oral glucose tolerance test as mentioned previously [43]), exclusion criteria – for the both groups – included heart failure, left ventricular systolic dysfunction (by ultrasound), congenital heart disease, significant valvular heart disease, acute coronary syndromes within previous 3 months, any surgery in past 6 months, infections within previous 2 months, arterial hypertension uncontrolled adequately by drugs, overt extracoronary atherosclerosis, relevant coexistent diseases, severely decreased estimated glomerular filtration rate (eGFR) (<30 mL/min per 1.73 m^2^ body-surface area according to the simplified equation developed by the Modification of Diet in Renal Disease study group [44]), other significant abnormalities in routine laboratory assays and any chronic non-CV medication as described earlier in detail [43,45]. Additionally, all the subjects were receiving an angiotensin-converting enzyme (ACE) inhibitor, statin and low-dose aspirin for ≥3 months prior to index hospitalization [43,45]. In agreement with the Declaration of Helsinki, the protocol had been approved by the university ethics committee and each patient gave informed consent.

### Biochemical assays

Peripheral venous blood sampling was performed 0–2 days before elective coronary angiography, plasma was separated from ethylenediaminetetraacetic acid-anticoagulated blood and frozen at −70°C until routine and extended biochemical assays. An index of IR was derived from homeostasis model assessment (HOMA-IR) as a product of fasting plasma insulin (μU/mL) and glucose (mmol/L) divided by 22.5 [46].

As described previously [43], plasma ADMA and L-arginine were determined by a commercially available enzyme-linked immunosorbent assays (ELISA) (DLD Diagnostika GmbH., Hamburg, Germany), previously validated against high-performance liquid chromatography coupled to mass spectrometry, the golden standard for ADMA determinations [47]. According to the manufacturer, the lower detection limit of ADMA assay was 0.05 μmol/L and intra-assay and inter-assay coefficients of variation averaged 7.5 and 10.3%, respectively. Cross-reactivity with L-arginine, symmetric dimethylarginine and *N*^*G*^-monomethyl-L-arginine was <0.02%, 1.2%, and 1.0%, respectively. With regard to L-arginine, the lower detection limit was 3.0 μmol/L, intra-assay and inter-assay coefficients of variation averaged 3.6% and 8.3%, respectively, and cross-reactivity was 0.01% for ADMA and 0.68% for symmetric dimethylarginine.

### Angiographic indices of CAD

Angiographic CAD was quantified by 3 different measures. First, we computed the number of vessels diseased, i.e., the number of major epicardial coronary arteries with ≥1 luminal diameter stenosis of ≥70% (≥50% for the left main coronary artery considered 3-vessel disease in a left dominant circulation and equivalent to an additional two diseased vessels in a right dominant or balanced circulation) as developed by Ringqvist et al. [48] on the basis of the Coronary Artery Surgery Study (CASS). Second, according to Sullivan et al. [49], a CAD extent score was calculated representing an approximate proportional length of the visible coronary vessels with irregularities of the vessel wall [45]. In case of inadequate collateralization of totally occluded vessels, the mean extent score of the vessel segments proximal to the occlusion was assigned to the occluded segment. Third, we computed Gensini score which reflects both CAD extent and severity assigning a heavier weight to the more severe luminal narrowings (by a non-linear point system) and to coronary segments serving larger regions of the myocardium [50]. All angiographic analyses were performed by a cardiologist blinded to clinical and biochemical characteristics.

### Statistical analysis

Data are shown as mean and SD for normally distributed continuous variables, median and interquartile range for not normally distributed values, and numbers with percentages for categorical characteristics. The accordance with a normal distribution was verified by the Lilliefors’ test and logarithmic transformation was applied when necessary. Intergroup comparisons were performed by 2-sided unpaired Student’s *t*-test or Fisher’s exact test. Trend effects according to the number of vessels diseased or over the quartiles of Gensini and Sullivan scores were estimated by Spearman’s rank-order correlation coefficient (ρ) for continuous values and the chi-squared test for trend for categorical data. Bivariate correlations were assessed by Pearson’s correlation coefficient (*r*). For multivariate approach, multiple linear regression was applied, including exclusively the covariates for which the univariate *P* value was ≤0.15.

Our study design allowed to detect a difference in mean ADMA concentrations and average HOMA-IR by 0.05 μmol/L and 0.8, respectively (about 0.46 SD), between the CAD subjects with 3-vessel disease compared to those with 1-/2-vessel CAD with a statistical power of 80% at a type I error rate of 0.05. Additionally, for the same power and type I error rate, bivariate correlations could be estimated with an *r* value of 0.225 in the CAD group as a whole.

Analyses were performed using STATISTICA (data analysis software system, version 10.0.1011.0; StatSoft, Inc., Tulsa, OK, USA). A *P* value <0.05 was inferred significant.

## Results

### Baseline patients’ characteristics according to angiographic CAD

Intergroup comparisons are presented in Table 1. The presence of significant coronary narrowings was associated with a lower concentration of high-density lipoproteins (HDL) cholesterol, marginally reduced eGFR, and a tendency to higher high-sensitivity C-reactive protein. In addition, ADMA levels were higher in patients with obstructive CAD compared to the controls (0.51 ± 0.10 vs. 0.46 ± 0.09 μmol/L, *P* = 0.01), whereas HOMA-IR was similar across the groups (median 3.2 [2.4–4.9] vs. 2.9 [2.3–4.7], *P* = 0.2) (Table 1).

**Table 1:** Baseline characteristics of CAD patients and controls

**Variable**	**CAD patients ( *n *= 151)**	**Controls ( *n *= 34)**	***P*value**
Age (years)	57 ± 11	56 ± 12	0.6
Body-mass index (kg/m^2^)	27.5 ± 3.8	27.3 ± 3.7	0.8
Current smokers, *n* (%)	41 (27%)	7 (21%)	0.5
Left ventricular ejection fraction (%)	69 ± 7	70 ± 6	0.4
Hypertension, *n* (%)	121 (80%)	25 (74%)	0.4
Systolic blood pressure (mm Hg)	130 ± 10	129 ± 12	0.6
Diastolic blood pressure (mm Hg)	79 ± 8	80 ± 9	0.5
Estimated GFR (mL/min per 1.73 m^2^)	71 ± 10	75 ± 13	0.05
Low-density lipoproteins cholesterol (mmol/L)	2.8 ± 0.7	2.6 ± 0.7	0.3
High-density lipoproteins cholesterol (mmol/L)	0.9 ± 0.3	1.0 ± 0.4	0.02
Triglycerides (mmol/L)	1.5 ± 0.8	1.4 ± 0.7	0.5
High-sensitivity C-reactive protein (mg/L)	1.8 (1.1–3.8)	1.6 (1.0–3.3)	0.07
Glucose (mmol/L)	5.8 ± 0.8	5.6 ± 0.7	0.25
HOMA-IR index	3.2 (2.4–4.9)	2.9 (2.3–4.7)	0.2
ADMA (μmol/L)	0.51 ± 0.10	0.46 ± 0.09	0.01
L-arginine (μmol/L)	70 ± 18	71 ± 17	0.7

Data are shown as mean ± SD, median (interquartile range) or *n* (%).

*Abbreviations:ADMA* asymmetric dimethylarginine, *CAD* coronary artery disease, *GFR* glomerular filtration rate, *HOMA-IR* homeostasis model assessment for insulin resistance.

Among the CAD patients, the proportions of subjects with 1-vessel, 2- vessel and 3-vessel CAD were 26%, 25% and 49%, respectively. The median Sullivan score was 29 (20–41) and Gensini score 32 (15–64). Indices of CAD extent and severity were associated with an older age (*P* = 0.01–0.10), depressed eGFR (*P* = 0.008–0.02), lower HDL cholesterol (*P* = 0.0.03–0.14) and a history of current smoking (*P* = 0.04–0.12).

### ADMA and HOMA-IR in relation to angiographic indices of CAD

ADMA levels increased across ascending quartiles of Sullivan score (ρ = 0.23, *P* = 0.004), but not with Gensini score (ρ = 0.12, *P* = 0.15) or the number of vessels involved (ρ = 0.08, *P* = 0.3) (Table 2). L-arginine levels did not differ between the subgroups irrespective of the criterion used for CAD quantification (*P* > 0.3).

**Table 2:** ADMA and HOMA-IR according to angiographic CAD indices

	**Number of vessels diseased**				
	**1-vessel CAD ( *n *= 40)**	**2-vessel CAD ( *n *= 37)**	**3-vessel CAD ( *n *= 74)**	***P*for trend**	
ADMA (μmol/L)	0.49 ± 0.10	0.52 ± 0.11	0.50 ± 0.10	0.3	
HOMA-IR	3.3 (2.5–5.0)	3.5 (2.6–5.2)	3.0 (2.0–4.6)	0.6	
	**Sullivan extent score**				
	1^st^ quartile ≤20	2^nd^ quartile 21–29	3^rd^ quartile 30–41	4^th^ quartile >41	***P*for trend**
ADMA (μmol/L)	0.48 ± 0.09	0.49 ± 0.08	0.52 ± 0.10	0.54 ± 0.11	0.004
HOMA-IR	3.0 (2.1–4.5)	3.1 (2.7–4.9)	3.5 (2.7–5.4)	3.1 (2.3–4.8)	0.2
	**Gensini score**				
	1^st^ quartile <15	2^nd^ quartile 15–32	3^rd^ quartile 33–64	4^th^ quartile >64	***P*for trend**
ADMA (μmol/L)	0.49 ± 0.10	0.51 ± 0.10	0.50 ± 0.11	0.52 ± 0.12	0.15
HOMA-IR	3.1 (2.1–4.6)	3.3 (2.6–5.1)	3.0 (2.2–5.2)	3.2 (2.5–4.8)	0.4

Data are shown as mean ± SD or median (interquartile range).

*Abbreviations:ADMA* asymmetric dimethylarginine, *CAD* coronary artery disease, *GFR* glomerular filtration rate, *HOMA-IR* homeostasis model assessment for insulin resistance.

**Figure 1:**
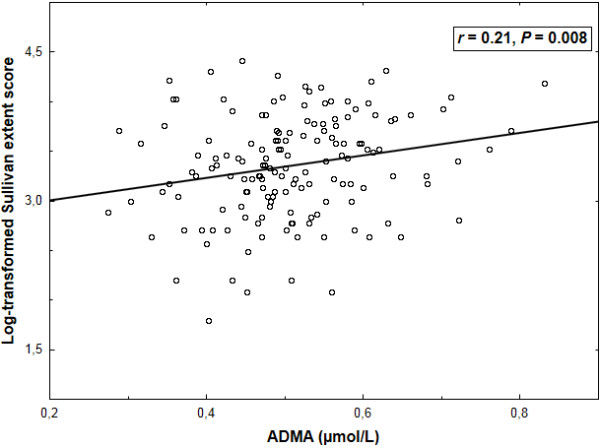
**A positive correlation between plasma levels of asymmetric dimethylarginine (ADMA) and log-transformed Sullivan score of the extent of coronary atherosclerosis.***r*, Pearson’s correlation coefficient.

ADMA correlated to log (Sullivan score) as a continuous variable (*r* = 0.21, *P* = 0.008) (Figure 1). By multiple linear regression with log-transformed Sullivan score as a dependent variable, the positive association of ADMA and log (Sullivan score) was only slightly attenuated upon adjustment for age, HDL cholesterol, eGFR and a history of current smoking (mean standardized *β*: 0.19 ± 0.08 [SEM], *P* = 0.015; adjusted *R*^2^: 0.08, *P* < 0.002).

HOMA-IR did not differ significantly according to any measure of angiographic CAD (*P* ≥ 0.2) (Table 2). ADMA and log-transformed HOMA-IR were unrelated (*r* = 0.07, *P* = 0.4).

## Discussion

Our salient finding was a rise in ADMA with increasing angiographic CAD extent but not with Gensini score or the number of major coronary vessels with a significant stenosis. Additionally, HOMA-IR was unrelated to either ADMA or any of the measures of angiographic CAD.

### ADMA versus angiographic CAD

That ADMA concentrations were higher in those with angiographic obstructive CAD compared to the controls free of significant CAD, is consistent with 3 out of 5 largest reports on ADMA in relation to angiographic CAD [7-10,14].

Meinitzer et al. [8] reported a univariate, but not multivariate, association of ADMA with Friesinger score in 2,543 participants of the Ludwigshafen Risk and Cardiovascular Health (LURIC) study (60% with stable CAD) with coronary stenoses ≥20%, nevertheless, Friesinger score is more strongly influenced by the severity and number of coronary narrowings than by CAD extent [51]. Lu et al. [10] observed higher ADMA with increasing maximal stenosis (<20% vs. 20–49% vs. ≥50%) in major coronary arteries by both univariate and multivariate approach in 997 patients with predominantly stable angina, which was yet limited to 638 non-diabetics. In 1,011 subjects undergoing elective coronary angiography, Wang et al. [9] reported elevated ADMA in the presence of ≥1 coronary stenosis of ≥50% and/or history of myocardial infarction or coronary revascularization. However, those authors [9,10] treated patients with a coronary diameter stenosis of ≥50% as a single group, whereas in the present study subjects with obstructive CAD were further discriminated according to the number of vessels involved and the Gensini and Sullivan scores.

On the other hand, in 1,364 patients, mainly non-diabetics with stable angina, Borgeraas et al. [14] recently reported even lower ADMA levels in subjects with significant CAD, which is contradictory to our findings. Nevertheless, they also observed no relationship between ADMA and the presence of 3-vessel CAD [14], in accordance with our data. Finally, as Schnabel et al. [7] did not list any measure of angiographic CAD among covariates associated with ADMA in 1,874 CAD patients from the Athero*Gene* study, it can possibly be suspected that those correlations were statistically insignificant. Similarly, Siegerink et al. [52] have not presented data on the relationship between angiographic variables and ADMA in 1,148 subjects with stable CAD participating in the KAROLA (“Langzeitfolge der KARdiOLogischen Anschlussheilbehandlung”) study.

Therefore, our findings suggest of a link between ADMA and diffuse, not focal, coronary atherosclerosis. As most acute coronary syndromes occur due to plaque rupture with superimposed occlusive thrombosis at sites involving non-obstructive coronary narrowings [53-56], this concept might explain significant independent associations of ADMA with adverse outcome but not with indices of CAD severity (the presence of angiographically obstructive CAD [9] or Friesinger score [8]) upon multivariate adjustment for traditional risk factors, renal function and C-reactive protein in the previously cited reports on patients referred to coronary angiography [8,9]. In keeping with this hypothesis, Bigi et al. [57] demonstrated that an angiographic extent score provided an additional prognostic information beyond the number of coronary lesions of ≥70% in 228 stable CAD subjects over a mean follow-up of 30 months. Furthermore, an association of elevated ADMA with the risk of myocardial infarction was found even by Borgeraas et al. [14] despite lower ADMA levels in subjects with significant CAD as mentioned previously.

### Insulin resistance versus angiographic CAD

The lack of correlation between HOMA-IR and angiographic CAD is contradictory to reports on a positive relationship between angiographic CAD extent and IR – assessed by the insulin suppression test or HOMA-IR – which was observed in small groups (*n* = 38–83) of non-diabetic subjects with stable CAD [23,24,26]. Additionally, we have previously found that the magnitude of integrated insulin response to an oral glucose tolerance test, a surrogate measure of IR [58], was related to multivessel CAD in 52 non-diabetic men below 50 years of age, which was absent in older subjects [59].

On the other hand, Vonbank et al. [28] observed no difference in HOMA-IR between subgroups of 986 consecutive patients undergoing elective coronary angiography divided according to the presence or number of lumen narrowings of ≥50%, irrespective of diabetes status. Accordingly, that the lack of a positive association between angiographic CAD and HOMA-IR was also reported in an adequately powered study [28], strengthens our negative findings. It is noteworthy that a negative result had been previously described in 797 men by Solymoss et al. [22] who related the number of coronary arteries with ≥50% stenosis to fasting insulinemia, a surrogate marker of IR. Although An et al. [29] identified HOMA-IR as an independent predictor of one-year coronary atherosclerosis progression in 366 CAD subjects, however, Gensini score at baseline did not correlate to HOMA-IR, in agreement with our results.

Nevertheless, a recent meta-analysis involving over 500,000 subjects without diabetes, mainly without prevalent atherosclerotic CV disease at baseline, proved the ability of HOMA-IR to predict incident CV events with a 46% increase in the relative risk of symptomatic CAD per 1-SD increment in HOMA-IR [19]. This was consistent with the independent association of HOMA-IR and CV mortality in 5,511 nondiabetic, adult participants of the third U.S. National Health and Nutrition Examination Survey (NHANES III) during a mean follow-up of 8.5 years [18]. Furthermore, Tenenbaum et al. [17] confirmed an independent adverse prognostic effect of HOMA-IR in 2,938 patients with preexisting CAD over a mean follow-up of 6.2 years. However, it is noteworthy that in 5,464 participants of Multi-Ethnic Study of Atherosclerosis (MESA) free of prevalent atherosclerotic CV disease the prognostic effect of HOMA-IR on the incidence and progression of coronary artery calcification over a 1.6–3.2-year follow-up disappeared after adjustment upon the components of the metabolic syndrome, lying downstream of IR [60].

Accordingly, it was hypothesized that the detrimental prognostic value of HOMA-IR in non-diabetic CAD patients even after controlling for the presence of the metabolic syndrome [61] or its traits [17,62] might rather be attributable to the precipitation of acute CV events than to the slow progression of coronary atherosclerosis [28]. In addition, a role of IR in the development of restenosis after coronary stenting has also been suggested [63,64]. Therefore, this concept is analogous to the previously proposed hypothesis suggestive of the mechanisms other than ADMA being a correlate of more severe coronary atherosclerosis as an explanation of the well-recognized ability of ADMA to predict adverse outcome in CAD [7-10,14].

### Insulin resistance in relation to the L-arginine–NO–ADMA pathway

In our hands, HOMA-IR and ADMA were independent of each other, confirming an earlier analysis in a subgroup of the study patients [43]. Stühlinger et al. [31] were the first to report a positive association between ADMA levels and the magnitude of IR in hypertensive and normotensive subjects, which precipitated the notion of ADMA as a common contributor to IR and endothelial dysfunction [65], an antecedent of coronary plaques [66,67]. In that seminal study IR was quantified as a steady-state plasma glucose concentration during simultaneous infusions of insulin, glucose and a somatostatin analogue, i.e., insulin suppression test [31]. Intriguingly, in spite of experimental evidence supporting mechanistic links between ADMA and IR within the liver and the skeletal muscle [68], the majority of clinical studies ‒ except for Marliss et al. [34] ‒ revealed no relationship of ADMA and the insulin-mediated glucose uptake during a hyperinsulinemic euglycemic clamp [30,35,69,70], the golden standard method for IR assessment. Additionally, inconsistent results were obtained when IR was represented by HOMA-IR or a steady-state plasma glucose during the insulin suppression test with either positive [32,33,38,42] or lacking [36,37,39] relations between IR and ADMA.

On the other hand, the magnitude of IR was linked to a polymorphism in the gene encoding one of isoforms of dimethylarginine dimathylaminohydrolase (DDAH-2) [40], an enzyme controlling the majority of ADMA catabolism [71]. In an animal study circulating ADMA, in contrast to endothelial function, was related to the activity of DDAH-1 but not DDAH-2 [72], the latter being predominant in the vascular endothelium [73]. Accordingly, the link between circulating ADMA and the degree of IR could be obscured by the inability of plasma ADMA to reflect its intracellular level in endothelial cells, which had previously been suggested by Maas et al. [74] as an explanation for the lack of correlation between plasma ADMA and polymorphisms in the promoter region of the DDAH-2 gene that were related to the prevalence of hypertension.

Irrespective of mutual relations between ADMA and IR, their differential associations with the generation and/or bioavailability of NO, an endogenous antiatherogenic molecule, appear relevant in terms of atherogenesis. Accordingly, that in our patients angiographic CAD extent was correlated to ADMA, but not IR, is compatible with the recent report by Tessari et al. [75] who demonstrated that ADMA but not IR was a negative modulator of whole-body NO generation in subjects with atherosclerotic risk factors. Admittedly, previous studies suggested a coupling of insulin metabolic sensitivity to insulin’s vascular effects and endothelial function [76-78]. However, although insulin-induced vasodilation is NO-dependent [76], there is also evidence supporting the dissociation between, on the one side, insulin’s ability to stimulate glucose uptake, and, on the other side, insulin’s vasodilatory action as well as NO formation or bioavailability both at baseline and under hyperinsulinemic conditions [30,79-81]. That Natali et al. [82] recently observed a deterioration in glucose tolerance but no changes in the magnitude of peripheral IR on acute inhibition of NO synthesis in non-diabetic subjects, is also consistent with the notion of IR-independent effects of NO deficiency.

Finally, in addition to the putative ability of ADMA to impair NO generation, other potential mechanisms of proatherogenic ADMA activity were recently suggested, such as an association of ADMA with increased arterial stiffness described in prediabetic subjects [83] and patients on maintenance hemodialysis [84]. Although peripheral blood pressure was similar in our CAD subjects and controls, however, elevated central arterial pressure in the presence of higher ADMA could have contributed to coronary atherosclerosis.

### Study limitations

First, the number of participants was relatively small and the number of subjects in the control group was over 4-fold lower compared to the CAD group. Nevertheless, we applied a wide set of exclusion criteria in order to restrict the sources of interindividual variability within our study groups. Second, ADMA levels can be influenced by medications, especially ACE inhibitors [85] and possibly some statins [73]. However, the ability of this effect to interfere with the associations of ADMA with angiographic CAD was probably limited because we had included exclusively patients who were receiving ACE inhibitors and statins for ≥3 months prior to blood withdrawal for ADMA assay. Third, the magnitude of IR was estimated by HOMA, whereas a hyperinsulinemic euglycemic clamp is the golden standard for IR quantification. Furthermore, Reaven [86] recently raised concerns with regard to using HOMA-IR as a measure of IR when altered insulin clearance considerably affects changes in fasting insulinaemia, which was shown on acute NO formation blockade [82]. This limitation constrains conclusions based on HOMA-IR in our previous study [43] and also the present one, all the more because interindividual differences in ADMA could influence NO bioavailability [87]. Nonetheless, for feasibility reasons, we were not able to perform the clamps in a series of CAD patients referred to diagnostic coronary angiography.

## Conclusions

In summary, ADMA levels appear to reflect diffuse but not focal coronary atherosclerosis in non-diabetic men with stable CAD irrespective of the magnitude of impaired metabolic sensitivity to insulin. Hence, the independent association between ADMA and coronary atherosclerotic burden may contribute to the well-recognized prognostic effect of ADMA in CAD.

## Abbreviations

ACE: Angiotensin-converting enzyme; ADMA: Asymmetric dimethylarginine; CAD: Coronary artery disease; CV: Cardiovascular; DDAH: Dimethylarginine dimethylaminohydrolase; eGFR: Estimated glomerular filtration rate; HDL: High-density lipoproteins; HOMA-IR: Homeostasis model assessment for insulin resistance; IR: Insulin resistance; NO: Nitric oxide.

## Competing interests

The authors declare that they have no competing interests.

## Authors’ contributions

OK conceived and designed the study, collected and analyzed data, and wrote the manuscript. AS and AG contributed to the study design, data analysis and discussion, and supervised the study. All authors read, critically revised and approved the final manuscript.
